# Vegetation Greening and Climate Change Promote Multidecadal Rises of Global Land Evapotranspiration

**DOI:** 10.1038/srep15956

**Published:** 2015-10-30

**Authors:** Ke Zhang, John S. Kimball, Ramakrishna R. Nemani, Steven W. Running, Yang Hong, Jonathan J. Gourley, Zhongbo Yu

**Affiliations:** 1 Cooperative Institute for Mesosacle Meteorological Studies, The University of Oklahoma, 120 David L. Boren Blvd., Norman, OK 73072, USA; 2 Hydrometeorology & Remote Sensing (HyDROS) Laboratory and School of Civil Engineering and Environmental Sciences, University of Oklahoma, Norman, OK; 3 State Key Laboratory of Hydrology-Water Resources and Hydraulic Engineering, Hohai University, 1 Xikang Road, Nanjing, Jiangsu Province, 210098, China; 4 Numerical Terradynamic Simulation Group, The University of Montana, 32 Campus Drive #1224, Missoula, MT 59812-1224, USA; 5 NASA Ames Research Center, Moffett Field, CA, USA; 6 NOAA/National Severe Storms Laboratory, Norman, Oklahoma 73072, USA; 7 Department of Hydraulic Engineering, Tsinghua University, Beijing, China

## Abstract

Recent studies showed that anomalous dry conditions and limited moisture supply roughly between 1998 and 2008, especially in the Southern Hemisphere, led to reduced vegetation productivity and ceased growth in land evapotranspiration (ET). However, natural variability of Earth’s climate system can degrade capabilities for identifying climate trends. Here we produced a long-term (1982–2013) remote sensing based land ET record and investigated multidecadal changes in global ET and underlying causes. The ET record shows a significant upward global trend of 0.88 mm yr^−2^ (*P* < 0.001) over the 32-year period, mainly driven by vegetation greening (0.018% per year; *P* < 0.001) and rising atmosphere moisture demand (0.75 mm yr^−2^; *P* = 0.016). Our results indicate that reduced ET growth between 1998 and 2008 was an episodic phenomenon, with subsequent recovery of the ET growth rate after 2008. Terrestrial precipitation also shows a positive trend of 0.66 mm yr^−2^ (*P* = 0.08) over the same period consistent with expected water cycle intensification, but this trend is lower than coincident increases in evaporative demand and ET, implying a possibility of cumulative water supply constraint to ET. Continuation of these trends will likely exacerbate regional drought-induced disturbances, especially during regional dry climate phases associated with strong El Niño events.

Land ET plays a vital role linking global water, energy, and carbon cycles, and is controlled by environmental factors and vegetation dynamics. The terrestrial water availability (TWA), defined as precipitation (P) minus ET, strongly influences the states of global ecosystems and human well-being, while the climatic water deficit (CWD), namely P minus potential ET (PET)^1^, quantifies the discrepancy between terrestrial water supply and atmospheric moisture demand, and impacts the distributions of hydrological regimes, climate zones, and vegetation^2^. Global warming and anthropogenic activities have altered global ET^3^,^4^, precipitation (P)^5^ and runoff^6^, suggesting a general intensification or acceleration of the global water cycle^7^,^8^. However, asynchronous changes in these components may lead to hydrological deficits and adverse ecological consequences. Comprehensive analysis of recent changes in land ET and their attribution in the context of variable climatic and ecological control factors, and natural climate oscillations are still lacking.

We applied an updated process-based land surface ET/heat fluxes algorithm^9^ to estimate global terrestrial ET and PET at 1/12° spatial resolution on a daily basis from 1982 to 2013, driven by satellite observations of photosynthetic canopy cover and surface meteorology inputs (see Methods and Supplementary Section 1). We separated the primary climatic controls to ET into three independent factors: demand, supply and energy, and analyzed changes in the three factors over the 32-year period (see Methods). Demand is quantified by a calculated potential ET rate that only depends on variabilities of air temperature, air vapor pressure, and wind speed, while supply and energy are determined by precipitation and solar radiation, respectively. All the three factors are normalized between 0 and 1. Details on the definitions and calculations of these factors are described in Methods. Finally, we obtained gauge and satellite based global monthly precipitation from three independent sources and used these data with the ET and PET records produced in this study to investigate changes in the global TWA and CWD over the 32-year period.

## Results

**Figure 1:**
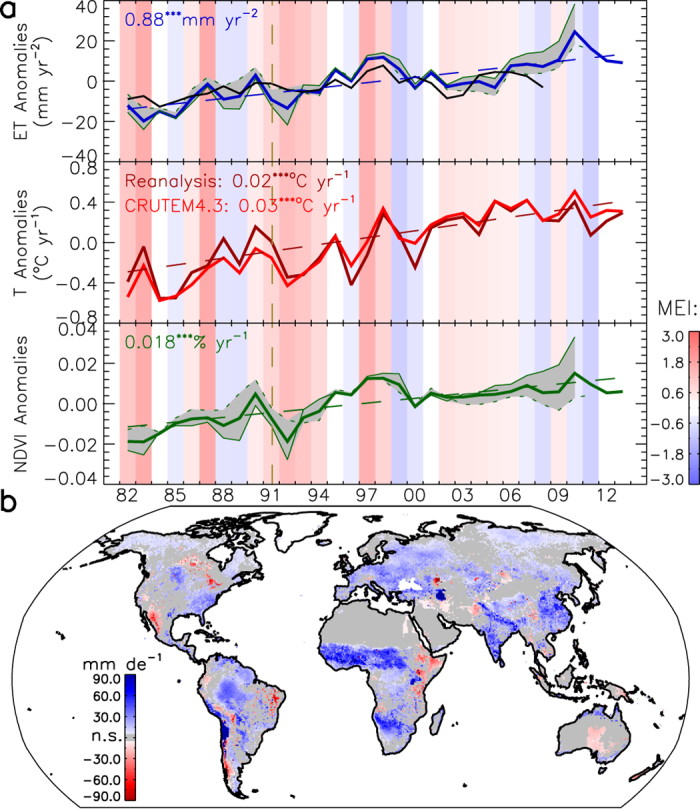
(**a**) Annual anomalies of remote sensing based global land ET estimates from 1982 to 2013 for the global land area (snow/ice covered areas excluded), global land air temperature and NDVI. The vertical brown dashed line indicates the June 1991 volcanic eruption of Mount Pinatubo. The grey area denotes the min-max ensemble range and provides a relative measure of uncertainty for the ET and NDVI due to differences between the third generation Global Inventory Modeling and Mapping Studies (GIMMS3g) and University of Arizona Vegetation Index and Phenology lab (VIP) NDVI time series. Another data-driven global land ET estimate from a previous study^3^ is shown as a black line. A multivariate ENSO index, MEI^48^, is shown with vertical color shading, where red and blue shades denote respective positive (El Niño) and negative (La Niña) phases, and darker shades indicate greater MEI intensity. (**b**) Spatial pattern of global land ET trends from 1982 to 2013; areas with non-significant (*P* ≥ 0.1) trends are marked in grey. The linear trends in (**a**,**b**) are calculated by the Kendall-Theil robust line and shown as dashed lines in (**a**). This figure was created using the Interactive Data Language (IDL) Core Version 7.1.2.

The domain of this study includes global land areas and inland water bodies that are not permanently covered by snow/ice, covering 89% (132.05 × 10^6^ km^2^) of the global land area. The multi-year mean of estimated global annual ET is 563.4 mm yr^−1^ (74.3 × 10^3^ km^3^ yr^−1^) with an inter-annual variability of 10.4 mm yr^−1^ (1.3 × 10^3^ km^3^ yr^−1^) from 1982 to 2013, which is similar to other reported global estimates^3^,^10^,^11^, considering differences in the extent of study areas. The modeled monthly ET compares favorably with global *in situ* tower measurements at the 1-km flux tower footprint scale and with independent ET estimates inferred from long-term water balance measurements from 284 globally distributed basins covering 65% of the global vegetated area (see Supplementary Information Section 2 and Fig. S1). It also agrees reasonably well with three independent ET data sets (see Supplementary Information Section 2 and Fig. S2). As a whole, the global land area shows a significant upward ET trend of 0.88 mm yr^−2^ (*P *<* *0.001) during the 32-year period (Fig. 1a). The upward trends occur widely across the globe with 42% of the global land area experiencing significant (*P *<* *0.1) positive trends in ET (Fig. 1b) and exceeding areas (13% of global land area) with significant negative trends (Fig. 1b). The rising global ET is consistent with the upward trend seen in a data-driven global ET record from 1982 to 2008 in a previous study^3^ (the black line in Fig. 1a). The global ET record derived in the current study shows similar inter-annual variability (r = 0.75; *P *<* *0.01) with the ET record in the previous study^3^ for the overlapping 1982–2008 period. Both the current results and ET records of the previous study^3^ show slowing or ceased growth rates from 1998 to 2008, which was primarily attributed to moisture limitation in the Southern Hemisphere^3^. However, our extended record shows a recovered ET growth rate after 2008 (Fig. 1a). This suggests that the lapse in the ET growth rate from 1998 to 2008 is likely an episodic phenomenon of the Earth’s climate system. This finding is also supported by another recent study, which suggests that the ET declines from 1998 to 2008 reflect transitions to El Niño conditions and are not the consequence of a persistent reorganization of the terrestrial water cycle^12^.

The upward trend of global ET coincides with positive trends in annual land surface air temperature from ground observations (CRUTEM4.3^13^: 0.03 °C yr^−1^; *P *<* *0.001) and the reanalysis record (0.023 °C yr^−1^; *P *<* *0.001), and satellite-observed ensemble-mean Normalized Difference Vegetation Index (NDVI) greenness (0.018% per year; *P *<* *0.001) (Fig. 1a). Global annual ET is highly correlated with NDVI (r* *= 0.91; *P *<* *0.001) and annual mean air temperature (r* *= 0.69; *P *<* *0.001). These parameters explain 84% of the variation in annual ET and have the similar positive trends as global annual ET, indicating that the global ET trend is mainly driven by increases in vegetation density and warming. A temporary global cooling and corresponding vegetation browning event occurred after the June 1991 Mount Pinatubo eruption^14^, resulting in a temporary decrease in ET (Fig. 1a). The El Niño-Southern Oscillation (ENSO) does not correspond strongly with variations of annual land-surface air temperature, NDVI, and ET at the global scale (Fig. 1a). This is likely due to complex, lagged responses of the entire troposphere to the ENSO activities^15^. In addition, the ENSO is associated with dry-wet cycles in many land areas^16^, leading to more complex ENSO linkages with vegetation states and vegetation-impacted ET.

**Figure 2:**
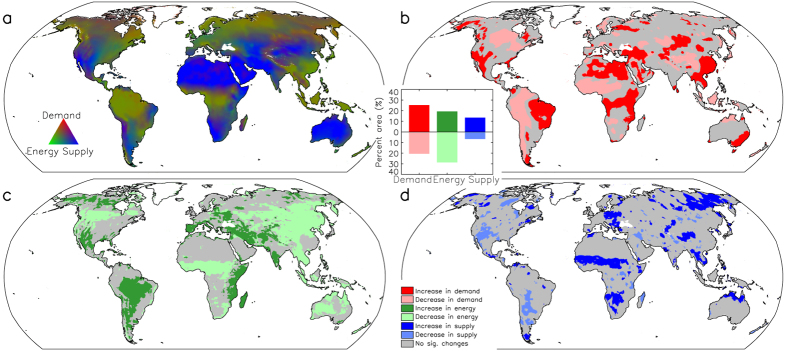
Geographic distribution of primary climatic control factors regulating terrestrial ET derived from multi-year (1982–2013) reanalysis and remote sensing based radiation data (map (a)). Global areas showing significant (*P *<* *0.1) changes from 1982 to 2013 in demand (**b**), energy (**c**) and supply (**d**) controls to ET are also shown. The inset graph shows the total proportional (%) areas experiencing significant trends in the three climatic control factors. White land areas denote persistent ice/snow cover and were masked from the analysis. This figure was created using the IDL Core Version 7.1.2.

We constructed a global map of relative contributions of the major climatic control factors (demand, supply and energy) influencing ET (see Methods). Water supply most strongly influences ET over 49% of the global domain, whereas available energy and atmospheric water demand are dominant influences on ET over 32% and 19% of the global domain, respectively (Fig. 2a). The atmospheric demand for ET shows the most extensive changes globally over the 32-year record (Fig. 2b). Approximately 29% of the global domain shows significant (*P *<* *0.1) increases in ET demand from 1982 to 2013, mainly driven by a general global warming trend and associated increases in air vapor pressure deficit (Supplementary Fig. S3), while 8% of the domain shows significant decreases in ET demand for the period, including western South America, and resulting from regional cooling and/or reduction of vapor pressure deficit (Supplementary Fig. S3e). The available energy for ET shows the second most extensive significant changes over the global domain (Fig. 2c). Approximately 8% of the domain shows significant increases in available energy for ET, mainly over tropical and subtropical regions (Fig. 2c) due to reduction in total cloudiness^17^; about 18% of the domain shows significant reductions in available energy for ET, mainly located over eastern Asia, central North America, Sudanian Savanna, and Australia (Fig. 2c), which are mainly caused by reduced incoming solar radiation over the study period due to emission of anthropogenic aerosols^18^. The available water supply for ET shows the smallest trends (Fig. 2d). Only small portions of the global domain show significant changes in water supply (9% and 6% for increasing and decreasing supply, respectively). Regions with significant positive water supply trends are mainly located in the subtropics and high latitudes, including the African Sahel, while areas with significant negative water supply trends are predominantly located in the mid-latitudes. The observed changes in climatic control factors predominantly favor increasing ET, consistent with the finding of rising global land ET over the same period. However, increases in water supply are lagging the coincident rise in atmospheric moisture demand with warming, indicating that ET may become more supply limited on a global basis if these trends persist in the future.

**Figure 3:**
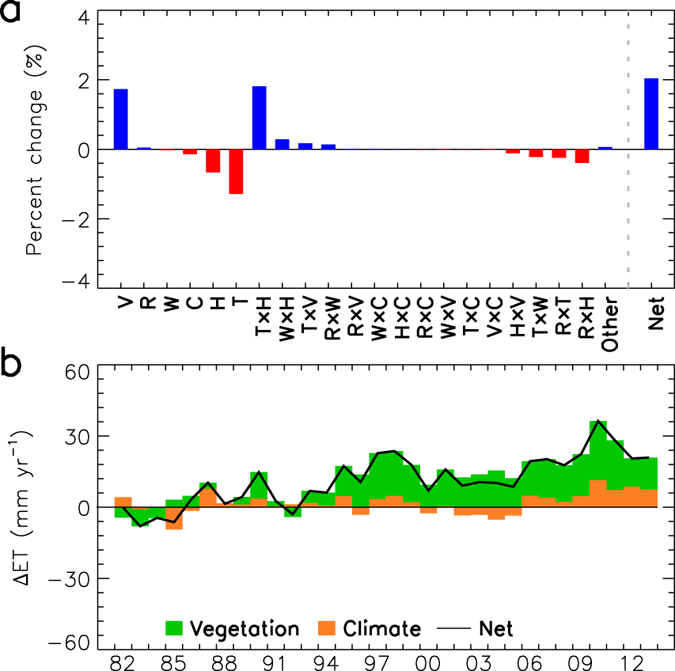
(**a**) Factorial contributions of vegetation dynamics (V), solar shortwave radiation (R), wind speed (W), atmospheric CO_2_ concentration (C), actual air vapor pressure (H), air temperature (T), and their two-way and higher-order interactions affecting global land ET; (**b**) yearly contributions of the most influential factor, V, and sum of the other factors affecting global land ET. This figure was created using the IDL Core Version 7.1.2.

The factorial analysis of variance to the ET estimates show that changes in vegetation greenness, air temperature and vapor pressure, and the two-way interactive effect of air temperature and vapor pressure are the dominant factors contributing to the multi-decadal changes in global ET (Fig. 3a). The direct effects of varying air temperature and vapor pressure have opposite impacts on ET relative to their two-way interactive effect (Fig. 3a), leading to a small net effect on ET. Changes in solar radiation impose a slight positive effect on ET, while wind speed and atmospheric CO_2_ exert slight negative effects on ET due to CO_2_ fertilization^19^ and recent declining wind speed^20^, respectively; however, these effects are predominantly outweighed by the impact of vegetation change (Fig. 3a). As a result, the net effect of climate change and CO_2_ fertilization is positive but much smaller than the effect of vegetation change (Fig. 3b), indicating that vegetation change is the primary driver of the global ET trend over the study period. Considering that vegetation change is also a response of the Earth’s terrestrial ecosystems to climate change, climate change imposes an indirect impact on ET by influencing global vegetation.

**Figure 4:**
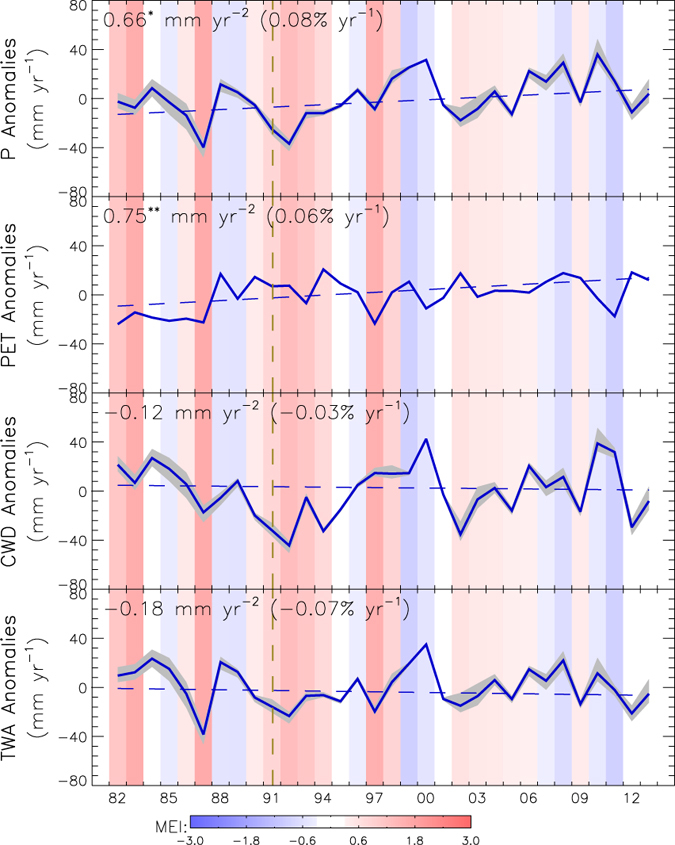
Yearly anomalies of P, PET, CWD (P-PET), and TWA from 1982 to 2013; the linear trends of annual values of the above three variables are calculated by the Kendall-Theil robust line and shown as dashed lines. Grey areas denote the min-max ensemble range as a relative measure of uncertainty in the global calculations. The color scheme of the MEI is the same as in Fig. 2.; *P* *<* *0.1, **P <* *0.05. This figure was created using the IDL Core Version 7.1.2.

To determine the combined impacts of solar radiation, air temperature, wind speed and air vapor pressure on atmospheric demand for ET, we calculated PET using the Penman equation and used this as a surrogate measure of atmospheric moisture demand. The resulting global PET record shows a strong, increasing trend of 0.75 mm yr^−1^ (*P *= 0.016) over the 32-year record (Fig. 4) coinciding with global warming and consistent with the exponential increase in atmospheric capacity to hold moisture with increasing temperature as defined by the well-known Clausius-Clapeyron relationship. Global P for the domain shows large temporal variability with a positive trend of 0.66 mm yr^−2^ (*P *= 0.08) (Fig. 4). The resulting CWD shows a weak, negative trend of −0.12 mm yr^−2^ (*P *= 0.83) (Fig. 4) and indicates a slight tendency to growing deficit between atmospheric moisture demand and available water supply for evaporation. This general increase in climate aridity is supported by a significant positive trend in global annual vapor pressure deficit (1.12 Pa yr^−1^; *P *= 0.02) over the same period (Supplementary Fig. S4). The TWA also shows a similar slight downward trend (−0.07% per year; *P *= 0.59) because a positive ET trend is offsetting increases in P over the 32-year record. This implies that any increases in P are being lost to ET rather than being allocated to other components of the global water cycle. The ENSO driven climate oscillations impart clear impacts to global P, indicated by significant negative correlation between the annual MEI (Multivariate ENSO Index) and annual P (*r *= −0.71; *P *<* *0.001). During strong, positive (i.e. El Niño) ENSO phases, the global land area as a whole receives less-than-normal P as shown in Fig. 4, despite that precipitation responses to El Niño events may differ in different regions. On the other hand, the global land area has lumped more-than-normal P during strong, negative (i.e. La Niña) ENSO phases (Fig. 4). Due to the close relationship between global P and ENSO activities, the global CWD and TWA are also correlated with ENSO (r* *= −0.43, *P *<* *0.01; r* *= −0.62, *P *<* *0.001) (Fig 4).

Natural ENSO-driven climate oscillations are associated with periodic wetting and drying cycles for terrestrial precipitation and are superimposed on a longer-term, positive P trend. If positive PET and ET trends continue to outpace growth in P (Fig. 4), ET and associated ecosystem processes will become more supply-limited, especially in these areas closely responding to “natural” El Niño dry cycles. Global climate projections also indicate future changes in ENSO characteristics and increasing occurrence of El Niño events^21^. These changes are likely to exacerbate global water deficits and the frequency, extent and severity of drought. Severe droughts serve as environmental triggers for other vegetation disturbances, including productivity decline^22^, mortality^23^,^24^, insect epidemics^25^,^26^ and fire^27^,^28^. These events may stimulate environmental tipping points toward large-scale ecosystem adjustments and biome conversions^1^,^29^ with continued climate warming.

## Methods

### ET Algorithm

The remote-sensing-driven ET algorithm used in this study is called Process-based Land Surface Evapotranspiration/Heat Fluxes algorithm (P-LSH). P-LSH quantifies canopy transpiration using the Penman-Monteith (PM) approach coupled with biome-specific canopy conductance determined from NDVI, soil evaporation through a modified PM approach, and open water evaporation using the Penman equation. The core components of this algorithm (i.e., the baseline algorithm) are described in Zhang *et al.*^30^. In this study, we made the following improvements to the baseline algorithm to account for the influence of variable wind speed and atmospheric CO_2_ concentrations on respective model aerodynamic conductance and canopy stomatal conductance terms, and resulting ET calculations: (1) quantify the impacts of increasing atmospheric CO_2_ concentrations on canopy stomatal conductance through a CO_2_ constraint function used in the MOSES land surface scheme^31^, (2) use surface wind speed to calculate aerodynamic conductance following Monteith and Unsworth^32^, and (3) replace the Priestley-Taylor method^33^ in the base algorithm with Penman equation^34^ to estimate open water evaporation and potential evaporation (PET) for considering the impacts of changes in surface wind speed on open water evaporation. More details on the improvements of this P-LSH algorithm are described in Supplementary Section 1. Although the P-LSH algorithm do not explicitly account for the effects of local perturbations, such as land use and irrigation, on ET, which may influence local ET estimates, it at least takes these effects into account to some extent from the satellite-observed NDVI data. Besides the vegetation dynamics (V) quantified by the NDVI data, the other forcing data of this algorithm include air temperature (T) and vapor pressure (H), wind speed (W), surface radiation fluxes (R), and atmospheric CO_2_ concentration (C).

### Data

The global datasets used in this study include daily surface meteorology records, satellite remote sensing data for driving the ET and PET algorithms, and precipitation and discharge records for the water balance analyses and ET validation. Daily meteorological data were from the NCEP/DOE AMIP-II Reanalysis (NCEP2)^35^. We verified the quality of surface meteorological inputs against measurements from ground observation networks and through intercomparison with two other meteorological reanalyses, and confirmed a generally good quality in these forcings (see Supplementary Section 4). Remote sensing radiative flux data, including clear-sky incoming solar shortwave radiation, all-sky downward solar shortwave radiation, and all-sky net shortwave solar radiation, were from the NASA World Climate Research Programme/Global Energy and Water-Cycle Experiment (WCRP/GEWEX) Surface Radiation Budget (SRB) Release-3.0 datasets (hereafter denoted as the SRB3.0) and the Clouds and the Earth’s Radiant Energy System (CERES) SYN1deg radiative fluxes. The SRB3.0 grid has a resolution of 1 degree latitude globally, and longitudinal resolution ranging from 1 degree in the tropics and subtropics to 120 degrees at the poles, while CERES SYN1deg has a resolution of 1° × 1°. We applied a statistical method to fuse the SRB3.0 data with CERES SYN1deg data by preserving the inter-annual variabilities and temporal trends of SRB3.0 on a grid cell-by-cell basis (see Supplementary Section 5). The other remote sensing inputs data include the 500-m MODIS-IGBP collection 5 global land cover classification^36^, 1-km global canopy height data^37^, 1-km AVHRR Tree Cover Continuous Fields data^38^, 1/12° semi-monthly Global Inventory Modeling and Mapping Studies (GIMMS3g) NDVI^39^, 0.05° bi-weekly University of Arizona Vegetation Index and Phenology lab (VIP) NDVI^40^, and 500-m MODIS NDVI. The GIMMS3g, VIP, and MODIS records are available from 1982–2011, 1982–2010, 2000-present, respectively. We applied the same fusion method for the radiation data to adjust the GIMMS3g and VIP records to match the MODIS record by preserving their inter-annual variabilities and temporal trends on a grid cell-by-cell basis (see Supplementary Section 5). The adjusted GIMMS3g and VIP records were combined with the MODIS record to produce a consistent long-term NDVI record as the input to derive biome-specific canopy conductance for our ET algorithm^9^. Global monthly precipitation data were derived from four independent sources, including Global Precipitation Climatology Project Version 2.1 (GPCP)^41^, Global Precipitation Climatology Centre Version 6.0 (GPCC)^42^, and Climate Research Unit TS3.22 (CRU) (http://www.cru.uea.ac.uk/cru/data/hrg/cru_ts_3.22) datasets. The global basin observed monthly river discharge data were compiled and provided by Dai *et al.*^43^.

### Factorial Experiments and Trend Analysis

As deterministic models like the ET algorithm used in this study don’t have stochastic element and relevant replications, a common method to assess the average contribution of each input factor (main effect) to the response variable is to compare the difference of response variable between the simulation with only one varied factor and the control simulation^44^,^45^,^46^. The control simulation (CONTROL) in this study is the simulation driven by the mean (i.e. the multi-year mean for individual day of the Julian days) environmental and vegetation conditions of the 1980’s (1982–1989). The ET result from the control simulation represents the expected value given the normal 1980’s environmental and vegetation conditions. For example, the main effects of *R* (E_R_) and *T* (E_T_) are:


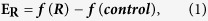


and


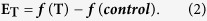


Following previous studies^45^,^47^, a two-way interaction between *R* and *T* (E_R×T_) is the subtraction of the main effects of R and T from the effect of the joint R plus T treatment and was calculated by:


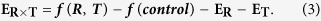


The other two-way interactive effects are calculated using the same methods. To derive the main and two-way interactive effects of all six factors, we conducted 23 sets of factorial simulations (summarized in Table 1): one control simulation driven by mean environmental and vegetation conditions of the 1980’s (1982–1989); six simulations where only one of the six factors is varied each time; and fifteen simulations where two of six factors were varied for each model run. The sum of all higher-order interactive effects is the difference between (*f*(*R,T,H,W,V,C*) − *f*(*control*)) and the sum of six main effects and fifteen two-way interactive effects.

All temporal trends in this study were estimated using the Kendall-Theil robust line and tested by the Mann-Kendall non-parametric test. Temporal anomalies were computed on a grid cell-by-cell basis and over the entire global domain relative to average conditions defined by the 32-year record. The null hypothesis of the trend testing is: there is no trend. The null hypothesis of the linear correlation testing is: there is no linear correlation between two variables. Statistical significance of the resulting trends was classified at α values of 0.1, 0.05, and 0.01. If a *P*-value is smaller than a given α, the null hypothesis is rejected at a significance level of α.

### Quantification of Primary Climatic Control Factors for ET

We combined the climatic factors affecting terrestrial ET into the three dominant environmental control factors; namely, demand, supply and energy. We quantified these three control factors by defining three dimensionless damping indices ranging from 0 (fully constrained) to 1 (no constraint) with decreasing constraints to ET. To calculate the demand multiplier, we first calculated annual PET (PET_FixedSW_) using the Penman equation driven by changing air temperatures and vapor pressure from NCEP2, with fixed daily shortwave radiation inputs from the fused SRB-CERES. We then calculated the multi-year average annual downward shortwave radiation (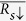) and the ratio of PET_FixedSW_ to 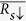. The ratio of PET_FixedSW_ to 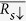 was then linearly scaled between 0 and 1 using the tenth and ninetieth percentiles of the resulting distribution. The scaled ratio of PET_FixedSW_ to 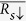 is defined as the demand multiplier, of which higher values coincide with higher atmospheric moisture demand. The ratio of annual P to multi-year mean annual PET_FixedSW_ (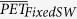) was scaled between 0 and 1, representing the tenth and ninetieth percentiles of the resulting variable distribution and is called the water supply multiplier. Higher and lower supply multiplier values indicate greater and lower potential moisture availability for ET, respectively. The ratio of all-sky to multi-year mean clear-sky downward shortwave radiation denotes the impacts of cloudiness and atmospheric constituents and the percentage of solar energy reaching the ground. This ratio was scaled between 0 and 1 according to the respective tenth and ninetieth percentiles of the resulting variable distribution and is defined as the energy multiplier.

## Additional Information

**How to cite this article**: Zhang, K. *et al.* Vegetation Greening and Climate Change Promote Multidecadal Rises of Global Land Evapotranspiration. *Sci. Rep.* **5**, 15956; doi: 10.1038/srep15956 (2015).

## Supplementary Material

Supplementary Information

## Figures and Tables

**Table 1:** Summary of all factorial simulations conducted in this study.

Simulation/Treatment^1^	Description
f(control)	Simulation with 1982–1989 mean (i.e. the multi-year mean for individual day of the Julian days) climate condition, atmospheric CO_2_ concentration and vegetation dynamics
*f(R)*	Radiation fluxes vary according to the SRB-CERES record; other variables according to control conditions
*f(T)*	Temperatures vary according to the NCEP2 record; other variables according to control conditions
*f(H)*	Air water vapor pressure varies according to the NCEP2 record; other variables according to control conditions
*f(W)*	Wind speed varies according to the NCEP2 record; other variables according to control conditions
*f(V)*	Vegetation varies according to the harmonized remote sensing NDVI record; other variables according to control conditions
*f(C)*	Atmospheric CO_2_ concentration varies according to the NOAA ESRL record; other variables according to control conditions
*f(R,T)*	Radiation fluxes and temperatures vary; other variables according to control conditions
*f(R,H)*	Radiation fluxes and air water vapor pressure vary; other variables according to control conditions
*f(R,W)*	Radiation fluxes and wind speed vary; other variables according to control conditions
*f(R,V)*	Radiation fluxes and vegetation vary; other variables according to control conditions
*f(R,C)*	Radiation fluxes and atmospheric CO_2_ concentration vary; other variables according to control conditions
*f(T,H)*	Temperatures and air water vapor pressure vary; other variables according to control conditions
*f(T,W)*	Temperatures and wind speed vary; other variables according to control conditions
*f(T,V)*	Temperatures and vegetation vary; other variables according to control conditions
*f(T,C)*	Temperatures vary and atmospheric CO_2_ concentration vary; other variables according to control conditions
*f(H,W)*	Air water vapor pressure and wind speed vary; other variables according to control conditions
*f(H,V)*	Air water vapor pressure and vegetation vary; other variables according to control conditions
*f(H,C)*	Air water vapor pressure and atmospheric CO_2_ concentration vary; other variables according to control conditions
*f(W,V)*	Wind speed and vegetation vary; other variables according to control conditions
*f(W,C)*	Wind speed and atmospheric CO_2_ concentration vary; other variables according to control conditions
*f(V,C)*	Vegetation and atmospheric CO_2_ concentration vary; other variables according to control conditions
*f(R,T,W,H,V,C)*	All variables vary

^1^The factors are: *R*, radiation fluxes; *T*, temperatures; *H*, air water vapor pressure; *W*, wind speed; *V*, vegetation; *C*, CO_2_.
