# Supplementary material for: Vegetation Greening and Climate Change Promote Multidecadal Rises of Global Land Evapotranspiration
Source: Sci Rep. 2015 Oct 30;5:15956. doi: 10.1038/srep15956 (PMC4626800; doi:10.1038/srep15956)
Supplement: Supplementary Information [file srep15956-s1.pdf]

SUPPLEMENTARY INFORMATION

Vegetation Greening and Climate Change Promote Multidecadal Rises of Global Land Evapotranspiration

Ke Zhang[1,2,3*], John S. Kimball[4], Ramakrishna R. Nemani[5], Steven W. Running[4], Yang Hong[2], Jonathan J. Gourley[6], and Zhongbo Yu[3]

[1]Cooperative Institute for Mesosacle Meteorological Studies, The University of Oklahoma, 120 David L. Boren Blvd., Norman, OK 73072, USA

[2]Hydrometeorology & Remote Sensing (HyDROS) Laboratory and School of Civil Engineering and Environmental Sciences, University of Oklahoma, Norman, OK

[3]State Key Laboratory of Hydrology-Water Resources and Hydraulic Engineering, Hohai University, 1 Xikang Road, Nanjing, Jiangsu Province, 210098, China

[4]Numerical Terradynamic Simulation Group, The University of Montana, 32 Campus Drive #1224, Missoula, MT 59812-1224, USA

[5]NASA Ames Research Center, Moffett Field, CA, USA

[6]NOAA/National Severe Storms Laboratory, Norman, OK

[*]Corresponding author: kezhang@ou.edu

1: Actual and Potential Evapotranspiration Algorithms

The remote-sensing-driven evapotranspiration (ET) algorithm used in this study is called Process-based Land Surface Evapotranspiration/Heat Fluxes algorithm (P-LSH), which quantifies canopy transpiration using the Penman-Monteith (PM) approach coupled with biome-specific canopy conductance determined from the Normalized Difference Vegetation Index (NDVI), soil evaporation through a modified PM approach, and open water evaporation using the Penman equation[1]. The core components of this algorithm are described in Zhang, et al. [1] (hereafter denoted as the baseline algorithm, ET_{base}). The ET results produced by the updated P-LSH are called ET_{RS}. In this study, we made the following improvements to the baseline algorithm to account for the influence of variable wind speed and atmospheric CO_2 concentrations on respective model aerodynamic conductance and canopy stomatal conductance terms, and resulting ET calculations:

(1) To quantify the impacts of increasing atmospheric CO_2 concentrations on canopy stomatal conductance, we applied a CO_2 constraint function to the biome-specific NDVI-based canopy conductance model of the base algorithm. The CO_2 constraint function follows the dependence of canopy conductance on CO_2 concentration used in the MOSES land surface scheme[2].

(2) We use surface wind speed information to calculate aerodynamic conductance (g_a: m s^{-1}) following Monteith and Unsworth[3]:

$$g_a = \frac{\kappa^2 u_m}{\ln\left[(z_m - d)/z_{om}\right]\ln\left[(z_m - d)/z_{ov}\right]} \quad (1)$$

where z_m(m) is the wind measurement height, which is set to canopy height in the calculation; d (m) is the zero plane displacement height; z_{om} and z_{ov} (m) are roughness lengths governing momentum transfer and the transfer of heat and vapor, respectively; κ (0.41) is the von Karman constant; u_m (m s^{-1}) is the wind speed at height z_m. We then calculate d, z_{om} and z_{ov} as functions of vegetation canopy height, h (m), following Allen et al.[4]:

$$d = \frac{2h}{3} \quad (2)$$

$$z_{om} = 0.123h \quad (3)$$

$$z_{ov} = 0.1z_{om} \quad (4)$$

Canopy heights were from a global 1-km canopy height database derived from spaceborne lidar[5].

(3) We used the Penman equation rewritten in metric units by Shuttleworth[6] to replace the Priestley-Taylor method[7] of the base algorithm to estimate open water evaporation and potential evaporation (PET). By using the Penman equation, we can account for the impacts of changes in surface wind speed on open water evaporation. We calculated the aerodynamic conductance term of the Penman equation using an estimation of 2-m height wind speed derived from the reanalysis data sets following Shuttleworth[6].

2. Validation of ET Estimates

The domain of this study includes global land areas and inland water bodies that are not permanently covered by snow/ice and defined by the 500-m MODIS collection 5 global land cover classification[8]. The total area considered in this study is about 132.05 $\times 10^6$ km^2 or 89% of the global land area. To validate our global ET estimates, we first compared the ET estimates using the updated P-LSH algorithm (ET_{RS}) at the 1-km scale with *in situ* tower eddy covariance measurements from 82 worldwide FLUXNET sites (Figure S1a). We also produced one additional set of ET estimates (ET_{Base}) at the site level using our previous ET algorithm, i.e., the baseline algorithm from Zhang et al.[1]. We then compared and evaluated relative accuracy of the two resulting ET series against the global tower observations. The ET_{RS} results show good agreement with the tower observations, indicated by a RMSE difference of 14.4 mm month^{-1} and R^2 value of 0.803 (Figure S1b), and are within the range of tower measurement uncertainty[9]. The ET_{RS} results showed better performance than the ET_{Base} (Table S1). Inclusion of the CO_2 regulation and dynamic aerodynamic conductance components reduced the RMSE difference of the resulting ET calculations by about 8% (1.2 mm month^{-1}) and increased the R^2 value by 0.08 (Table S1), suggesting that changing atmospheric CO_2

concentrations and seasonal and inter-annual variability in wind speed contribute to the variation in land surface ET estimates. We further compared the ET_{RS} results with independent ET estimates inferred from long-term water balance measurements ($ET_{Inferred}$) from 284 globally distributed basins covering 65% of the global vegetated area (Figure S1c). These results also show similar accuracy for the ET_{RS} calculations, with an estimated RMSE difference of 172.2 mm per year and R^2 of 0.810 (Figure S1d).

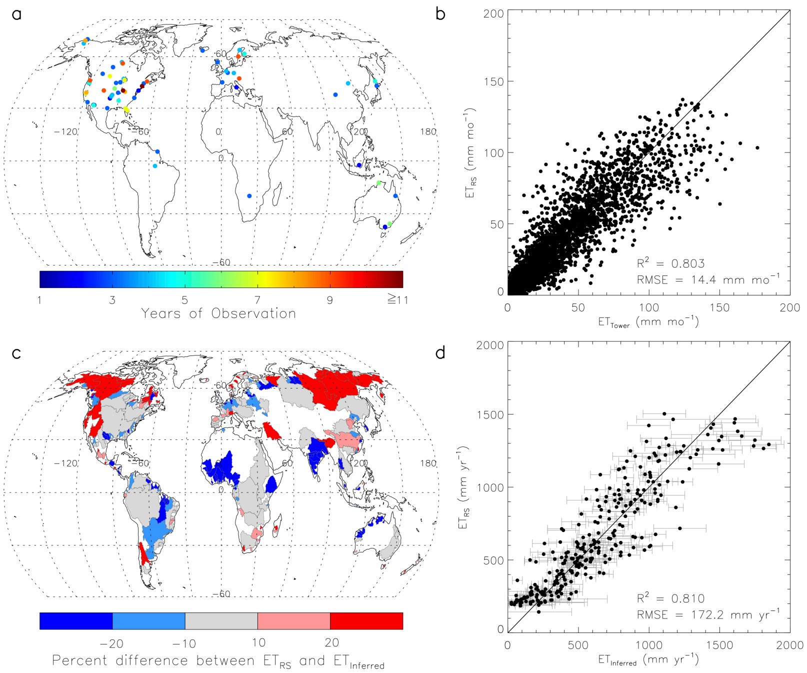

Figure S1. Validation of remote sensing based ET estimates (ET_{RS}) against independent observations at the site and basin levels. (a) Locations of 82 FLUXNET sites used for the ET validation; the color code refers to the number of years of observation at each site. (b) Comparison of monthly ET_{RS} against 1-km tower footprints and associated ET measurements at the 82 FLUXNET sites (n=3344 site-months) for the period of record 1992-2006. (c) Percent difference between multi-year (1983-2006) average ET_{RS} and inferred ET ($ET_{Inferred}$) from basin-scale multi-year water balance calculations for 284 major global basins. (d) Scatter plot of ET_{RS} vs. $ET_{Inferred}$ (n=284); error bars denote the min-max range of $ET_{Inferred}$ resulted from differences in the global precipitation datasets. This figure was created using the IDL Core Version 7.1.2.

Table S1. Performance statistics of two sets of ET simulations against independent measurements from 82 FLUXNET sites. The ET_{Base} simulations don't account for temporal variability in wind speed (WS) and resulting aerodynamic conductance, and impact of atmospheric CO_2 concentration on stomatal conductance, while the ET_{RS} simulations account for these effects.

**Method**	**MAE** (mm mo^{-1})	**RMSE** (mm mo^{-1})	R^2
ET_{RS}	9.7	14.3	0.803
ET_{Base}	10.8	15.5	0.795

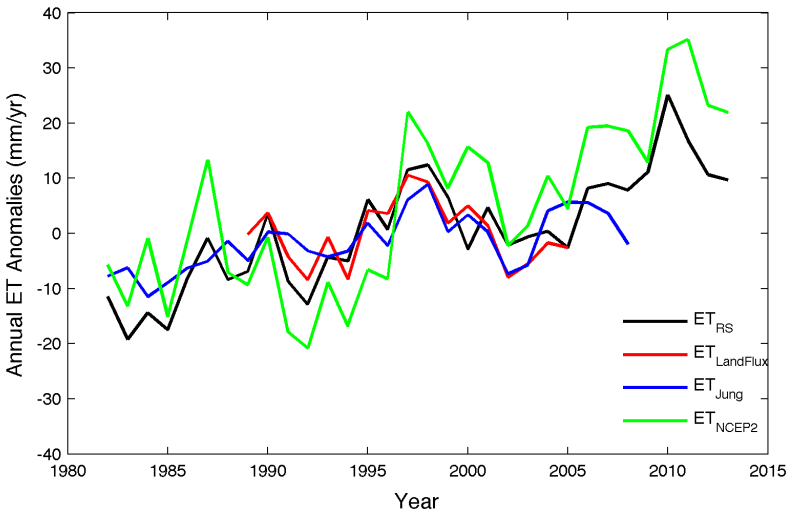

Figure S2. Annual anomalies of global land ET from this study (ET_{RS}), the ensemble median of fourteen independent global ET data sets by the LandFlux-EVAL project[*], a data-driven land ET record (ET_{Jung}), and the NCEP2 reanalysis (ET_{NCEP2}). This figure was created using MATLAB R2014a.

Furthermore, we compared our global land ET results with three independent ET data, including a data-driven land ET record[10] (ET_{Jung}), the NCEP2 reanalysis[11] (ET_{NCEP2}), and the ensemble median of fourteen independent global ET data sets provided by the LandFlux-EVAL project ($ET_{LandFlux}$)[12]. The fourteen independent global ET data sets collected by the LandFlux-EVAL project cover a wide range of data sources, including estimates from five diagnostic models, five land surface models, and four reanalyses[12]. However, the four reanalyses do not include the NCEP2 reanalysis. ET_{Jung} is available from 1982 to 2008, while $ET_{LandFlux}$ is only available from 1989 to 2005. ET_{NCEP2} is available for the whole study period. Our global ET results agree well with these three ET records in terms of inter-annual variability and

[*] Because the LandFlux-EVAL project (http://www.iac.ethz.ch/groups/seneviratne/research/LandFlux-EVAL) doesn't release the fourteen individual ET data sets, we cannot plot the inter-quartile range of the anomalies of the ensemble.

multidecadal trend (Figure S2). In particular, it matches better with ET_{Jung} and $ET_{LandFlux}$ than ET_{NCEP2} (Figure S2), suggesting that our ET data is likely more accurate than the ET data from the NCEP2 reanalysis. ET_{Jung} and $ET_{LandFlux}$ can be regarded as high-quality ET records or a reference data set because the former is an observation-based product and the latter is the ensemble median of fourteen independent ET data sets. This concludes that the inter-annual variability and temporal trend seen in our ET results are supported by other independent ET data.

3. Spatial Patterns of Changes in Meteorological Forcings and Their Temporal Trends from 1982 to 2013

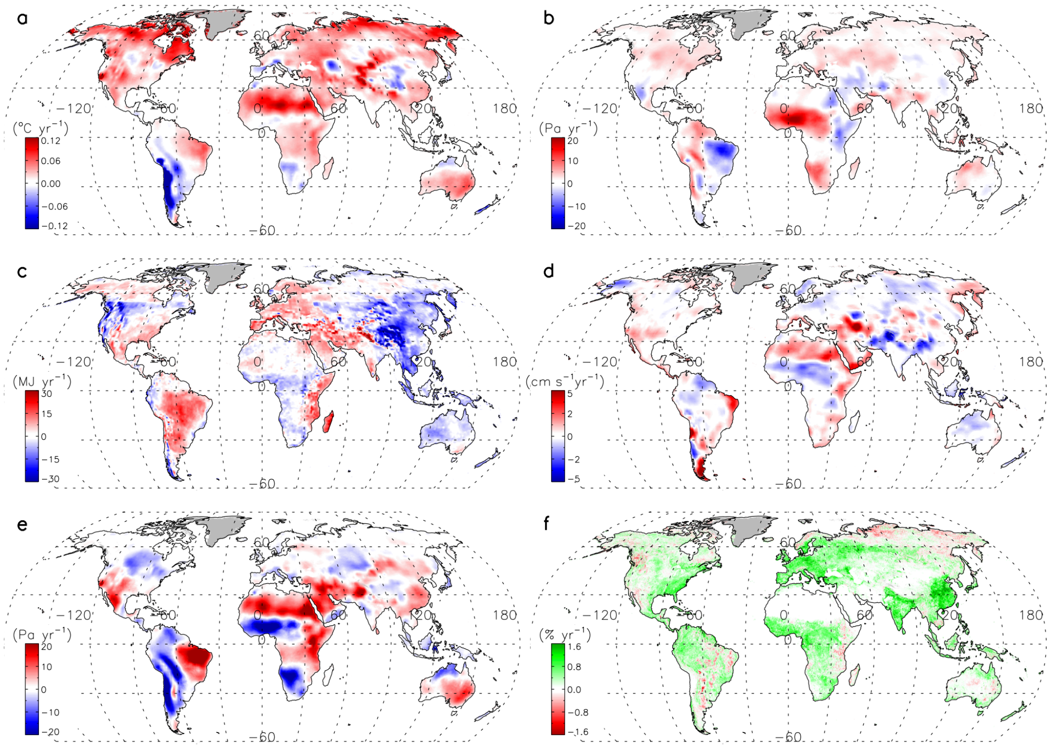

Figure S3. Spatial patterns of trends in annual average air temperature (T) (a), air vapor pressure (e_a) (b), annual total downward shortwave solar radiation ($R_{s\downarrow}$) (c), annual average 10-m wind speed (WS_{10}) (d), air vapor pressure deficit (VPD) (e), and annual average NDVI (f) from 1982 to 2013. The T, e_a, VPD and WS_{10} data were derived from NCEP2 reanalysis, whereas the $R_{s\downarrow}$ data were derived from the fused SRB-CERES record. NDVI data were obtained from harmonized AVHRR GIMMS3g, University of Arizona VIP, and MODIS records. This figure was created using the IDL Core Version 7.1.2.

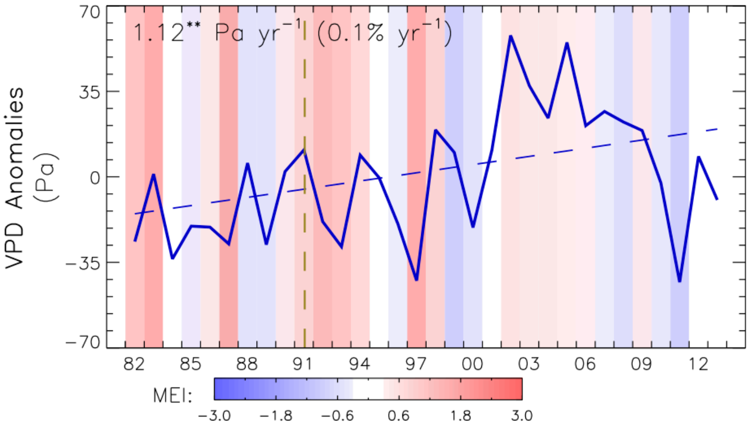

Figure S4. Yearly anomalies of air vapor pressure deficit (VPD) from 1982 to 2013; dashed line denotes the linear trend and Mann-Kendall test of trend significance. A multivariate ENSO index[13] is shown with vertical color shading, where red and blue shades denote respective positive (El Niño) and negative (La Niña) phases. ** $P < 0.05$. This figure was created using the IDL Core Version 7.1.2.

4. Evaluation of Global Meteorological Datasets

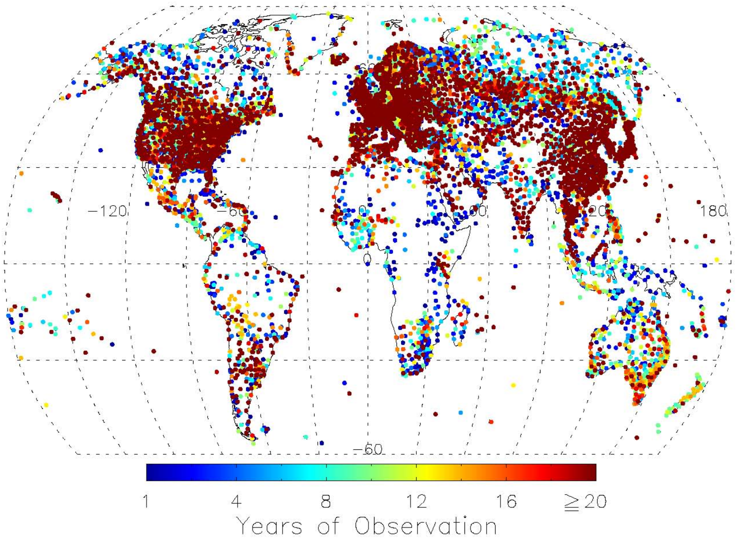

Figure S5. Locations of 11,322 WMO weather stations used for validation of daily meteorological forcings used in the global ET calculations; the color code refers to the number of years of observation between 1983 and 2006 at each station. This figure was created using the IDL Core Version 7.1.2.

To verify the quality of surface meteorological inputs, we evaluated the NCEP2 daily air vapor pressure (e_a), maximum air temperature (T_{max}), minimum air temperature (T_{min}), average air temperature (T_{avg}), and wind speed (WS) records against corresponding daily measurements of these parameters from the 11,322 WMO weather stations (Figure S5) provided by the National Climatic Data Center Climate Services

Branch (NCDC CSB; http://www.ncdc.noaa.gov) Global Summary of the Day Version 8. The total number of WMO stations used for these comparisons varied annually, ranging from 4,574 to 7,262. The WMO stations weren't used for evaluating the SRB-CERES solar radiation fluxes because these station records do not contain detailed solar radiation information.

We also compared T_{max}, T_{min}, T_{avg}, and e_a from NCEP2 and net solar radiation (R_n: W m^{-2}) obtained from the SRB-CERES record with daily measurements from 82 worldwide FLUXNET sites[9] (Figure S1a). The record lengths of these tower sites from 1983 to 2006 vary between 1 and 15 years (Figure S1a). A detailed summary of the flux tower sites are provided in Zhang, et al. [1]. The tower sites generally provide more complete and extensive records of R_n than the other solar radiation components. The R_n term also represents the net radiant flux from R_{so}, $R_{s\downarrow}$, and R_{ns} component fluxes and directly impacts the ET/latent heat flux. We therefore directly compare R_n of SRB-CERES with corresponding tower based R_n measurements. We didn't compare NCEP2 WS records with tower measurements because of incomplete WS records at the tower sites.

We used the following validation procedure to evaluate the relative accuracies of the meteorological reanalysis inputs in relation to surface measurements of these parameters. Error from the validation procedure is defined as the reference measurement (i.e., from *in situ* flux tower sites and weather stations) minus the estimated value defined from NCEP2 and SRB-CERES reanalysis records. The mean ($\bar{\varepsilon}$), median (M_{ε}), first quartile (Q_1), and third quartile (Q_3) of the resulting error distributions are used to assess error in the meteorological reanalysis datasets. The mean absolute error (MAE) and root mean square error (RMSE) were calculated to describe the spread of error, while the Pearson simple correlation coefficient (r) was used to evaluate the correspondences between the reanalysis datasets and station measurement records.

Comparisons between the NCEP2 record and daily meteorological measurements from the 11,322 WMO weather stations (Figure S5) show high accuracies and narrow error spreads in the reanalysis temperature and e_a variables (Table S2). These results indicate that NCEP2 provides high-quality temperature and e_a records. However, Table S2 results show that the daily WS records from NCEP2 have relatively low

correspondence with the WMO station observations, with a correlation of 0.571 (P < 0.001). The reduced correspondence for wind speed is not unexpected, because local wind conditions are spatially heterogeneous and are likely to be beneath the scale of variability represented by the coarse (1-2.5 degree) resolution NCEP2 reanalysis. The reanalysis wind speeds may also deviate from the *in situ* measurements because of differences between estimated wind profiles and local measurement heights; the NCDC station records don't provide measurement height information for wind speed so a 2-m measurement height was assumed for every site, whereby WS was estimated by power law extrapolation of 10 m height reanalysis wind speed records. The above assumptions and simplifications may cause large errors in the estimates of site-level 2-m wind speed.

Table S2. Summary of accuracy in the NCEP2 climate data by comparing with independent daily meteorological measurements from 11,322 WMO weather stations (Figure S5). All correlation coefficients (r) are significant with 99% confidence.

Variables	Source	$\bar{\varepsilon}$	Q_1	M_ε	Q_3	MAE	RMSE	r
	NCEP1	1.78	0.53	1.70	3.07	2.33	3.03	0.963
T_{max} (^{o}c)	NCEP2	1.91	0.67	1.77	3.10	2.35	3.08	0.964
	NCEP1	1.18	-0.03	0.93	2.14	1.74	2.53	0.968
T_{avg} (^{o}c)	NCEP2	0.75	-0.45	0.47	1.71	1.59	2.40	0.966
	NCEP1	0.55	-1.15	0.37	1.96	2.09	2.89	0.953
T_{min} (^{o}c)	NCEP2	-0.23	-1.76	-0.37	1.04	1.96	2.73	0.956
	NCEP1	-82.70	-160.56	-88.32	-10.34	138.75	184.49	0.964
e_a (Pa)	NCEP2	-43.06	-116.19	-46.58	23.41	117.46	172.82	0.962
	NCEP1	0.57	-0.26	0.52	1.32	1.07	1.42	0.553
WS (m s^{-1})	NCEP2	-0.55	-1.37	-0.61	0.18	1.09	1.40	0.571

In addition, NCEP2 have comparable accuracies for air temperature and vapor pressure variables ($T_{\max}$, T_{avg}, $T_{\min}$ and e_a) relative to daily measurements from the 82 flux towers (Table S3). The NCEP2 temperature variables show strong correspondence with the tower observations (r ≥ 0.948; P < 0.001). The e_a records from NCEP2 also correspond strongly with the tower observations, while the SRB-CERES R_n record has a

relatively lower, but favorable (r > 0.87; P < 0.001) correlation with the observations. The error distributions for the temperature variables are within 3.17-4.22 °C (RMSE), with a mean bias near 0.0°C. The NCEP2 e_a record shows a RMSE difference of 332.45 Pa relative to the station network measurements; this difference is approximately equivalent to the e_a variation produced from the air temperature RMSE difference. The SRB-CERES record shows slight overestimation of R_n relative to the tower observations with a RMSE of 34.11 W m^{-2}. The generally close correspondence between the SRB-CERES record and worldwide tower observations for R_n indicate that the remote sensing based radiation products provide a relatively accurate R_n record that is suitable for the global ET calculations.

Table S3. Summary of accuracy in the NCEP2 and SRB-CERES climate data by comparing with independent daily meteorological measurements from 82 FLUXNET sites (Figure S1a). All correlation coefficients (r) are significant with 99% confidence.

Variables	Source	$\bar{\varepsilon}$	Q_1	M_ε	Q_3	MAE	RMSE	r
T_{max} (°c)	NCEP2	0.06	-2.15	0.24	2.40	3.00	4.01	0.955
T_{avg} (°c)	NCEP2	-0.45	-2.11	-0.42	1.27	2.33	3.17	0.970
T_{min} (°c)	NCEP2	-1.08	-3.30	-1.13	1.10	3.17	4.22	0.948
e_a (Pa)	NCEP2	97.72	-58.01	54.38	228.70	212.74	332.45	0.936
R_n (W m^{-2})	SRB	-1.85	-20.59	-4.01	13.90	23.82	34.11	0.876

There is increasing evidence that the amount of solar radiation incident at the Earth's surface is not stable and undergoes significant decadal variations[14-17]. Wild[15] reviewed the evidence for these changes, their magnitude, possible causes, their representation in climate models and the potential implications of this solar variability for climate change. Wild[15] concluded that the various studies analyzing long-term records of surface radiation measurements suggest a widespread decrease in surface solar radiation between the 1950s and 1980s ("global dimming"), with a more recent partial recovery ("brightening") at many locations. However, Alpert et al.[18] showed that the "global dimming" phenomenon between the 1950s and 1980s is significantly dominated by large urban sites, suggesting that solar dimming, mainly caused by anthropogenic aerosols, happened at the local or regional scale and may not effectively represent global changes

in solar illumination. Pinker et al.[19] reported consistencies and differences in global solar radiation trends from 1983 to 2001 between satellite observations and ground measurements. These differences reflect the impacts of limited coverage of ground measurements on assessment of changes in global total solar radiation[19]. Relative to the ground measurements, the satellites can provide complete global coverage[19]. Hinkelman et al.[20] compared the SRB downward shortwave radiation data with measurements from Global Energy Balance Archive sites and showed favorable agreement between the SRB record and ground measurements, indicated by a respective root-mean-square difference and correlation of 2.6 W m^{-2} and 0.822. Betts et al.[21] compared NCEP2, SRB and ERA40 shortwave radiation fluxes and found that the major anomaly signals are similar in the two reanalysis data sets (NCEP2 and ERA40) and the SRB record despite biases in their respective climatologies.

Besides evaluating the NCEP2 reanalysis against the extensive site-level data, we also conducted intercomparison between the NCEP2 reanalysis, two other reanalysis products, the ERA-Interim[22] and MERRA[23] reanalysis data, and an observation-based surface air temperature dataset (CRUTEM4.3)[24]. When we compared the NCEP2 data with the CRUTEM4.3 data, we first aggregated the NCEP2 data to 5°, the spatial resolution of the CRUTEM4.3 data, so that the two series are comparable; when we compared the three reanalysis data, all reanalysis data were first downscaled to a common 1° resolution to ensure these comparisons comparable. On the global scale, the inter-annual variability and temporal trend of the NCEP2 air temperature are similar to those of the CRUTEM4.3, ERA-Interim, and MERRA air temperatures (Figure S6a). The correlation coefficients between any two of the four global annual air temperature records are larger than 0.96 ($P < 0.001$), highlighting that the four air temperature series are consistent with each other. The inter-annual variability and temporal trend of the NCEP2 global air vapor pressure also agrees well with those of the other two reanalyses as shown in Figure S6b and indicated by the high correlation coefficients ($r \geq 0.80$; $P < 0.001$). The NCEP2 global surface wind speed series shows a similar inter-annual variability to the ERA-Interim series (Figure S6c); both series show weak temporal trends. However, the MERRA wind speed series show a different trend relative to the NCEP2 and ERA-INTERIM series.

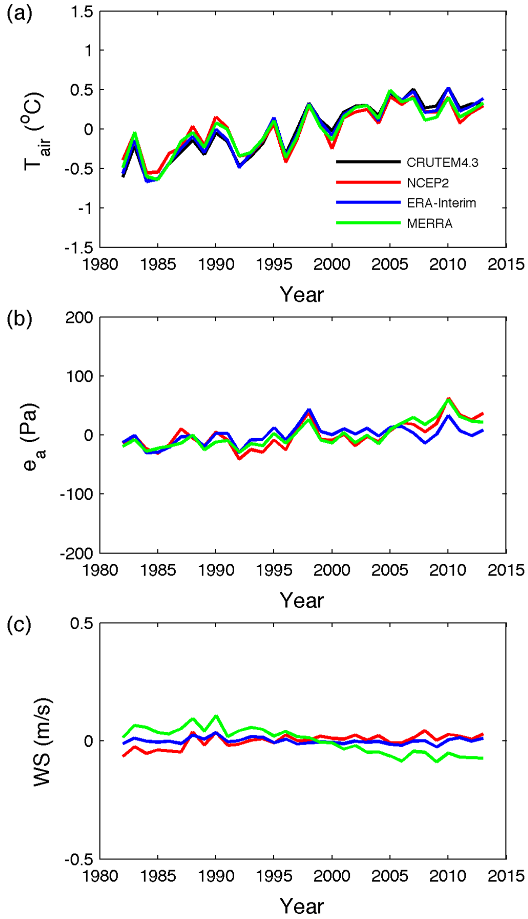

Figure S6. (a) Annual anomalies of global average land air temperature from the CRUTEM4.3, NCEP2, ERA-Interim, and MERRA data sets, (b) annual anomalies of global average air vapor pressure from the NCEP2, ERA-Interim, and MERRA data sets, and (c) annual anomalies of global average surface wind speed from the NCEP2, ERA-Interim, and MERRA data sets. This figure was created using MATLAB R2014a.

We also investigated the correspondence between these reanalysis and observation-based data sets on a grid cell-by-cell basis. The NCEP2 reanalysis show consistent inter-annual variabilities in air temperature with the CRUTEM4.3 record and the other two reanalysis data over a majority of land areas (Figure S7). This indicates that the NCEP2 air temperature is generally consistent with the other four data sets. It is also worth noting that there are relatively low consistencies in air temperature between the NCEP2 and the other data sets in some regions such as western Central America and Central Africa (Figure S7a-c). These low consistencies also appear in the comparison between the ERA-Interim and MERRA series (Figure S7d). This suggests that larger

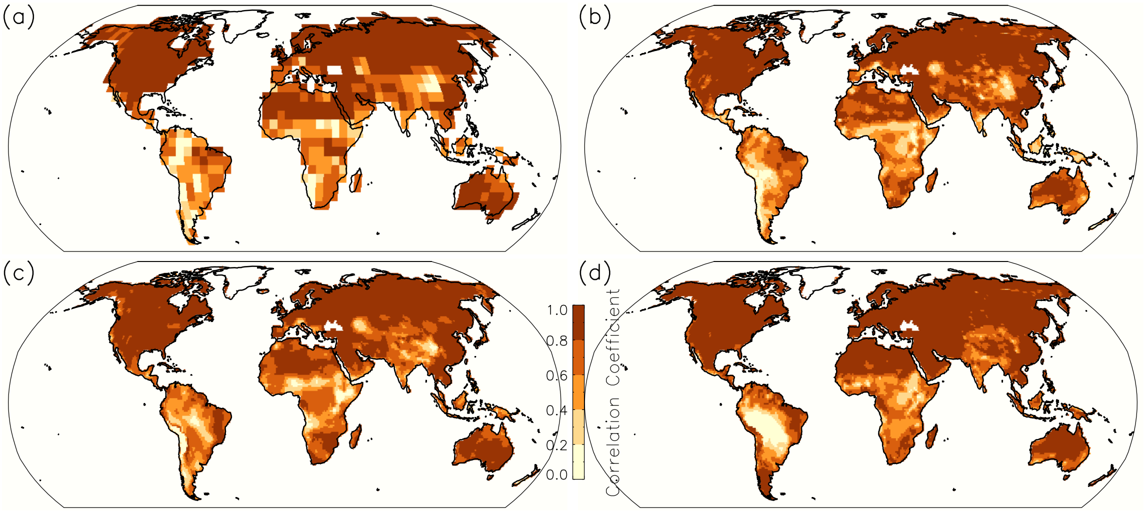

Figure S7. Correlation maps (a) between NCEP2 and CRUTEM4.3 annual air temperature series, (b) between NCEP2 and ERA-Interim annual air temperature series, (c) between NCEP2 and MERRA annual air temperature series, and (d) between ERA-Interim and MERRA annual air temperature series from 1982 to 2013. Because the original spatial resolution of the CRUTEM4.3 data is 5°, the NCEP2 was first aggregated to 5° before it was compared against the CRUTEM4.3 data. This figure was created using the IDL Core Version 7.1.2.

uncertainties may exist in these regions in all reanalysis data sets. These uncertainties are probably due to low availability of observations that can be used to improve the reanalyses through data assimilation in these regions. Compared to air temperature, air vapor pressure and surface wind speed show lower consistency across the three reanalysis data sets (Figures S8, S9). The lowest consistencies in the air vapor pressure and wind speed variables across all of these reanalysis sets mainly appear in South America, Africa and Central Asia (Figures S8, S9), suggesting that the air vapor pressure and wind speed variables in all of the three reanalyses may have larger uncertainty and that none of the three reanalysis sets show better qualities than the other sets according to these intercomparison.

In summary, the intercomparison between the NCEP2 reanalysis and three other reanalysis or observation-based data sets show that air temperature, air vapor pressure, and surface wind speed in the NCEP2 reanalysis show generally reasonably good agreements with the other meteorology data sets in most of global land areas. Although larger uncertainty exist in the NCEP2 reanalysis in some regions, the similar level of

uncertainty seems to appear in the other reanalysis sets as well. In other words, the three meteorological variables in the NCEP2 reanalysis are comparable to their counterparts in the ERA-Interim and MERRA reanalysis data.

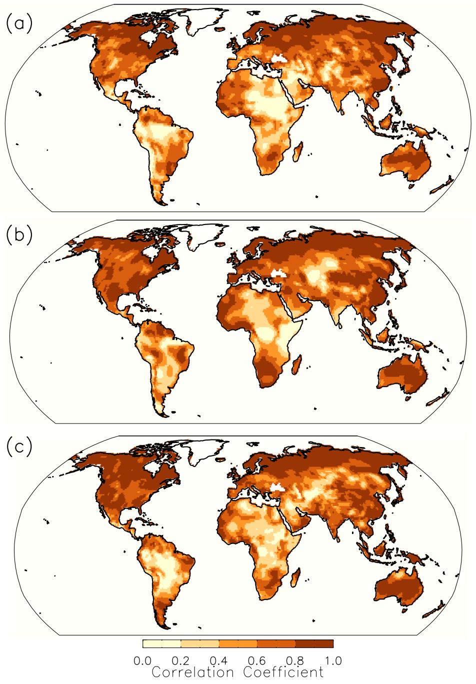

Figure S8. Correlation maps (a) between NCEP2 and ERA-Interim annual air vapor pressure series, (b) between NCEP2 and MERRA annual air vapor pressure series, and (c) between ERA-Interim and MERRA annual air vapor pressure series from 1982 to 2013. This figure was created using the IDL Core Version 7.1.2.

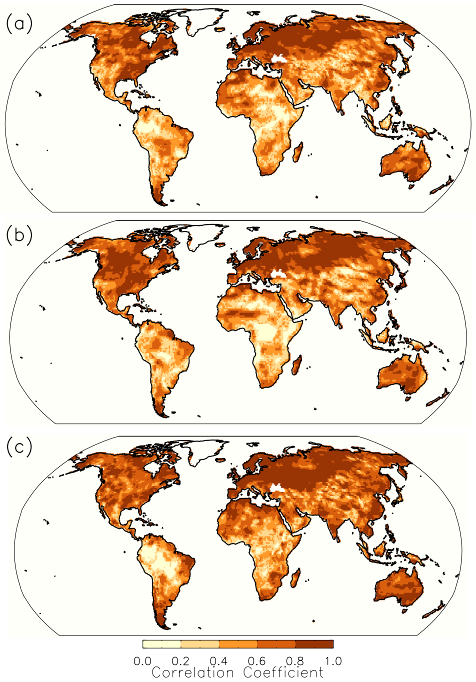

Figure S9. Correlation maps (a) between NCEP2 and ERA-Interim annual wind speed series, (b) between NCEP2 and MERRA annual wind speed series, and (c) between ERA-Interim and MERRA annual wind speed series from 1982 to 2013 This figure was created using the IDL Core Version 7.1.2.

5. Fusion of Data Records and Its Evaluation

Since there is not a single remote sensing based dataset providing the solar radiation fluxes and NDVI records for our whole study period (1982-2013), we had to fuse the SRB3.0 (available from 1982 to 2007 and at a spatial resolution of ~1°×1°) and CERES SYN1deg (available from 2000 to 2013 and at a spatial resolution of 1°×1°) data to produce a harmonized solar radiation dataset, and fuse the GIMMS3g (available from 1982 to 2011) and VIP (available from 1982 to 2010) NDVI records with the MODIS (available since 2000) NDVI to produce consistent NDVI datasets. The SRB3.0 grid has a resolution of 1 degree latitude globally, and longitudinal resolution ranging from 1 degree in the tropics and subtropics to 120 degrees at the poles. Before the SRB3.0 data were adjusted, we first re-gridded it to a 1°×1° grid through the bilinear interpolation method to match with the CERES SYN1deg data.

We adopted a sophisticated statistical method called equidistant cumulative distribution function (EDCDF) matching method[25] to do the data fusion. This method needs three CDFs: for the benchmark data (*BEN*) during the overlapping period of the benchmark data and the data to be adjusted, for the data to be adjusted during the overlapping period (MOD_o), and for the data to be adjusted during the non-overlapping period (MOD_n). The basic idea is to adjust the CDF of MOD_o ($\boldsymbol{F_{MOD_o}}$) to make $\boldsymbol{F_{MOD_o}}$ match with the CDF of BEN ($\boldsymbol{F_{BEN}}$), and to find the adjustment function (transfer function) based on the difference (Δ) between $\boldsymbol{F_{MOD_o}}$ and $\boldsymbol{F_{BEN}}$ for a given percentile of $\boldsymbol{F_{MOD_n}}$. For a value $\boldsymbol{x}$ in the data to be adjusted during the non-overlapping period, this method can be expressed as

$$\Delta = \boldsymbol{F_{BEN}^{-1}}\left(\boldsymbol{F_{MOD_n}}(\boldsymbol{x})\right) - \boldsymbol{F_{MOD_o}^{-1}}\left(\boldsymbol{F_{MOD_n}}(\boldsymbol{x})\right) \quad (5)$$

$$\boldsymbol{x_{adj}} = \boldsymbol{x} + \Delta \quad (6)$$

where $\boldsymbol{x_{adj}}$ is the adjusted value of $\boldsymbol{x}$ and $\boldsymbol{F^{-1}}$ is the inverse function of CDF. For the overlapping period,

$$\boldsymbol{x_{adj}} = \boldsymbol{F_{BEN}^{-1}}\left(\boldsymbol{F_{MOD_o}}(\boldsymbol{x})\right) \quad (7)$$

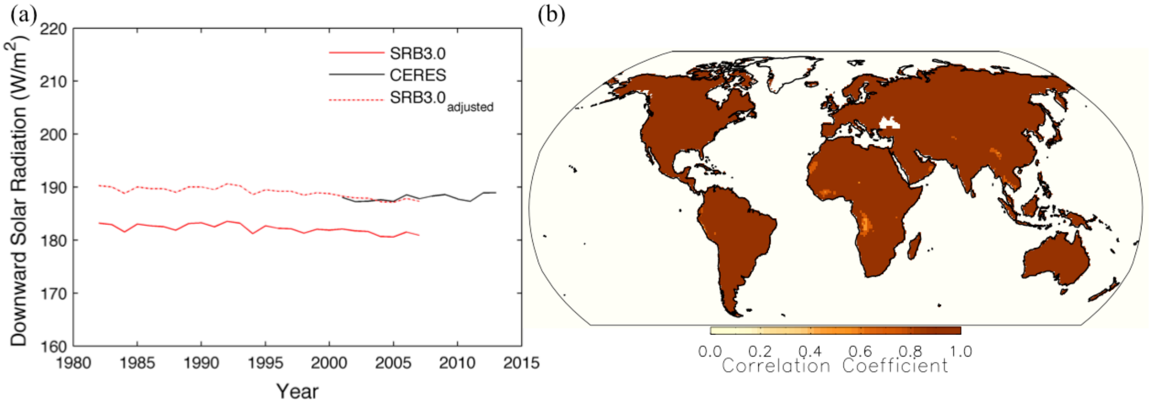

Figure S10. (a) Annual global average downward solar radiations from the CERES SYN1deg, original SRB3.0, and adjusted SRB3.0 records, and (b) correlation map between the original and adjusted SRB3.0 series. This figure was created using the IDL Core Version 7.1.2.

In this study, we treated the CERES SYN1deg and MODIS data as the benchmark and adjusted the SRB3.0 data to produce the fused SRB-CERES record. It is clear that

the original SRB3.0 downward shortwave radiation has systematically lower values than the CERES SYN1deg data (Figure S10a). Once the SRB3.0 data are adjusted using the above fusion method, the adjusted global annual SRB series match well with the CERES SYN1deg data while it still shows the same inter-annual variability as the original series indicated by a high correlation coefficient ($r = 0.99$; $P < 0.001$) (Figure S10a). The cell-by-cell correlation analysis also shows that this fusion method successfully preserves the trends and inter-annual variabilities in the original SRB3.0 data across the globe (Figure S10b).

We adjusted the GIMMS3g and VIP data to produce the adjusted GIMMS and VIP records. The original global average annual GIMMS3g and VIP data show systematically higher values than the MODIS data (Figure S11a). The applied fusion method successfully adjusted the GIMMS3g and VIP data to match with the MODIS data. Moreover, the correlation coefficients between global mean annual GIMMS3g NDVI series prior and after the adjustment, and between global mean annual VIP NDVI series prior and after the adjustment are 0.98 and 0.99, respectively, indicating the fusion procedure preserves the inter-annual variabilities of the original NDVI records. The cell-by-cell correlation analysis also shows that the original and adjusted GIMMS3g and VIP data shows high consistency across the globe in terms of inter-annual variabilities and trends (Figure S11b,c). We finally computed the mean of the adjusted GIMMS, adjusted VIP, and MODIS records as the final fused NDVI record for the 1982-2013 period.

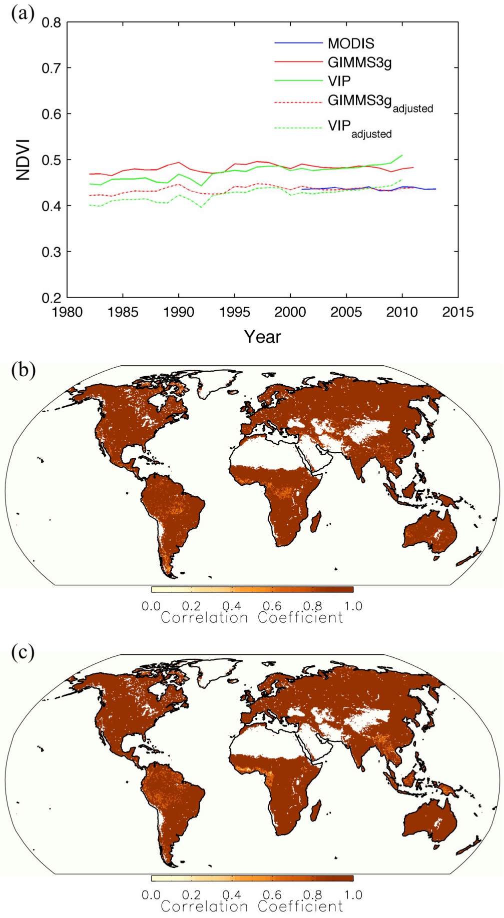

Figure S11. (a) Annual global mean NDVI values from the MODIS, GIMMS3g, VIP, adjusted GIMMS3g, and adjusted VIP records, (b) correlation map between the original and adjusted GIMMS3g series, and (c) correlation map between the original and adjusted VIP series. This figure was created using the IDL Core Version 7.1.2.

References

1. Zhang, K., Kimball, J. S., Nemani, R. R. & Running, S. W. A continuous satellite-derived global record of land surface evapotranspiration from 1983 to 2006. *Water Resour. Res.* **46**, W09522, doi:10.1029/2009WR008800 (2010).
2. Cox, P. M. *et al.* The impact of new land surface physics on the GCM simulation of climate and climate sensitivity. *Clim. Dyn.* **15**, 183-203 (1999).
3. Monteith, J. & Unsworth, M. *Principles of environmental physics*. (Academic Press, 2007).
4. Allen, R. G., Pereira, L. S., Raes, D. & Smith, M. *Crop evapotranspiration: Guidelines for computing crop requirements, FAO irrigation and drainage paper 56*. (Food and Agricultural Organization of the U.N., 1998).

5 Simard, M., Pinto, N., Fisher, J. B. & Baccini, A. Mapping forest canopy height globally with spaceborne lidar. *J. Geophys. Res.* **116** (2011).

6 Shuttleworth, W. J. in *Handbook of hydrology* (ed D.R. Maidment) 4.1-4.53 (McGraw Hill, 1993).

7 Priestley, C. H. B. & Taylor, R. J. On the assessment of surface heat flux and evaporation using large-scale parameters. *Mon. Weather Rev.* **100**, 81-92 (1972).

8 Friedl, M. A. *et al.* MODIS Collection 5 global land cover: Algorithm refinements and characterization of new datasets. *Remote Sens. Environ.* **114**, 168-182 (2010).

9 Baldocchi, D. Breathing of the terrestrial biosphere: lessons learned from a global network of carbon dioxide flux measurement systems. *Aust. J. Bot.* **56**, 1-26 (2008).

10 Jung, M. *et al.* Recent decline in the global land evapotranspiration trend due to limited moisture supply. *Nature* **467**, 951-954, doi:Doi 10.1038/Nature09396 (2010).

11 Kanamitsu, M. *et al.* NCEP-DOE AMIP-II Reanalysis (R-2). *Bull. Am. Meteorol. Soc.* **83**, 1631-1643 (2002).

12 Mueller, B. *et al.* Benchmark products for land evapotranspiration: LandFlux-EVAL multi-data set synthesis. *Hydrol. Earth Syst. Sci.* **17**, 3707-3720 (2013).

13 Wolter, K. & Timlin, M. S. Measuring the strength of ENSO - how does 1997/98 rank? *Weather* **53**, 315-324 (1998).

14 Andronova, N., Penner, J. E. & Wong, T. Observed and modeled evolution of the tropical mean radiation budget at the top of the atmosphere since 1985. *J. Geophys. Res.* **114**, D14106 (2009).

15 Wild, M. Global dimming and brightening: A review. *J. Geophys. Res.* **114**, D00D16 (2009).

16 Wild, M. *et al.* From dimming to brightening: Decadal changes in solar radiation at Earth's surface. *Science* **308**, 847-850 (2005).

17 Gilgen, H., Roesch, A., Wild, M. & Ohmura, A. Decadal changes in shortwave irradiance at the surface in the period from 1960 to 2000 estimated from Global Energy Balance Archive Data. *J. Geophys. Res.* **114**, D00D08, doi:10.1029/2008jd011383 (2009).

18 Alpert, P., Kishcha, P., Kaufman, Y. J. & Schwarzbard, R. Global dimming or local dimming?: Effect of urbanization on sunlight availability. *Geophys. Res. Lett.* **32**, L17802, doi:10.1029/2005gl023320 (2005).

19 Pinker, R. T., Zhang, B. & Dutton, E. G. Do satellites detect trends in surface solar radiation? *Science* **308**, 850-854 (2005).

20 Hinkelman, L. M., Stackhouse, P. W., Wielicki, B. A., Zhang, T. P. & Wilson, S. R. Surface insolation trends from satellite and ground measurements: Comparisons and challenges. *J. Geophys. Res.* **114**, -, doi:10.1029/2008jd011004 (2009).

21 Betts, A. K., Zhao, M., Dirmeyer, P. A. & Beljaars, A. C. M. Comparison of ERA40 and NCEP/DOE near-surface data sets with other ISLSCP-II data sets. *J. Geophys. Res.* **111**, D22S04, doi:10.1029/2006jd007174 (2006).

22 Dee, D. P. *et al.* The ERA-Interim reanalysis: configuration and performance of the data assimilation system. *Quart. J. Roy. Meteor. Soc.* **137**, 553-597, doi:10.1002/qj.828 (2011).

23 Reichle, R. H. *et al.* Assessment and Enhancement of MERRA Land Surface Hydrology Estimates. *J. Clim.* **24**, 6322-6338 (2011).

24 Jones, P. D. *et al.* Hemispheric and large-scale land-surface air temperature variations: An extensive revision and an update to 2010. *J. Geophys. Res.* **117** (2012).

25 Li, H. B., Sheffield, J. & Wood, E. F. Bias correction of monthly precipitation and temperature fields from Intergovernmental Panel on Climate Change AR4 models using equidistant quantile matching. *J. Geophys. Res.* **115**, D10101, doi:10.1029/2009jd012882 (2010).
